# Probe-Based Confocal Laser Endomicroscopy Using Acrinol as a Novel Dye Can Be Used to Observe Cancer Nuclei of Bladder Carcinoma *In Situ*

**DOI:** 10.1089/cren.2017.0114

**Published:** 2018-02-01

**Authors:** Yoshio Naya, Natsuki Takaha, Takako Okubo, Koji Shiota, Issei Hayashi, Masaru Mori, Seiki Date, Tsuneharu Miki, Osamu Ukimura

**Affiliations:** ^1^ Department of Urology, Meiji University of Integrative Medicine, Nantan, Japan.; ^2^ Department of Urology, Nagahama City Kohoku Hospital, Nagahama, Japan.; ^3^ Department of Pathology, Meiji University of Integrative Medicine, Nantan, Japan.; ^4^ Department of Urology, Saiseikai Shiga Hospital, Ritto, Japan.; ^5^ Department of Urology, Kyoto Prefectural University of Medicine, Kyoto, Japan.

**Keywords:** endomicroscopy, laser optical fiber, acrinol, carcinoma *in situ* of bladder

## Abstract

***Background:*** Cystoscopy using white light is a standard procedure for diagnosing bladder cancer; however, white light can result in missed lesions that are present, but not visible, such as in cases of carcinoma *in situ* (CIS). In this case report, we describe observing the nuclei of urothelial carcinoma cells *in situ* that were not visible with cystoscopy under white light using probe-based confocal laser endomicroscopy (pCLE) with acrinol and fluorescein during transurethral resection of a bladder tumor (TURBT).

***Case Presentation:*** A 59-year-old male with a medical history of neurogenic bladder dysfunction with multiple bladder diverticula was referred to the urology department for gross hematuria. TURBT was performed with the assistance of pCLE, using acrinol as a novel dye. Standard cystoscopy under white light could not detect any bladder tumor; however, pCLE using acrinol could detect the abnormal nuclei of bladder CIS. Subsequent histopathologic analysis of the specimen confirmed a diagnosis of bladder CIS. To our knowledge, this is the first reported case of bladder CIS diagnosed with the assistance of pCLE using acrinol in a patient undergoing a TURBT.

***Conclusion:*** pCLE using acrinol as a novel dye can help observe the cancerous nuclei of bladder CIS that cannot be detected using conventional cystoscopy under white light. Therefore, pCLE using acrinol is one possible modality for performing an optical biopsy during TURBT.

## Introduction and Background

Recently, previous reports have highlighted the utility of probe-based confocal laser endomicroscopy (pCLE) using fluorescein for detection of urothelial carcinoma (UC).^1,2^ Fluorescein revealed tissue structures of the UC without imaging the cell nucleus or cytoplasm.^1,2^ Meanwhile, a 0.02% acriflavine solution was able to stain the cell nuclei in gastric cancer^3^; however, acriflavine is harmful to the eyes and mucosa if inhaled and is irritable to the skin. A safer compound is ethacridine lactate (acrinol), which is a type of acridine derivative. Furthermore, it is currently used as an antiseptic agent for skin disinfection. We previously reported the feasibility of acrinol for pCLE to observe the nuclei of the UC cells *ex vivo*.^4^ In this case report, we describe observing the nuclei of UC cells of carcinoma *in situ* (CIS) that were not visible with cystoscopy under white light using pCLE with acrinol and fluorescein during transurethral resection of a bladder tumor (TURBT). This study was approved by the ethics committees of Meiji University of Integrative Medicine (Approval No.: 28-17 Meiji University of Integrative Medicine).

## Presentation of Case

A 59-year-old Japanese male presented to the outpatient clinic of Meiji University of Integrative Medicine with a chief complaint of asymptomatic gross hematuria.

He has mental retardation and neurogenic bladder dysfunction. Cystoscopy using white light showed multiple bladder diverticula and mild inflammation, but no tumors. The residual urine in the bladder was 200 mL. Urine cytology was class V. CT and bladder MRI could not detect any tumor within the urinary tract. After placement of a 16F Foley catheter into the bladder, 50 mL of a 0.1% acrinol solution and 1 mL of 10% fluorescein in 99 mL of normal saline were injected into the bladder using a 60-mL catheter-tip syringe. After 5 minutes, contrast agents were drained from the bladder using a 60-mL catheter-tip syringe and residual contrast agents were washed out from the bladder using 300 mL of normal saline.

**FIG. 1.**
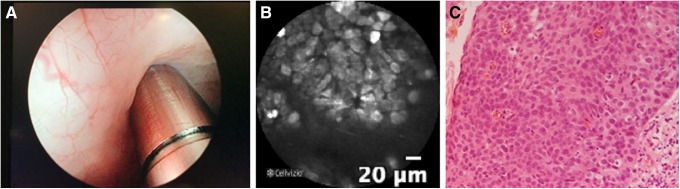
Representative images from white light cystoscopy and pCLE dependent on intravenous administration of acrinol and fluorescein. **(A)** White light cystoscopy with irregular mucosa in a bladder diverticulum. The 2.7 mm imaging probe is visible at the bottom of the image. **(B)** pCLE of intravesically stained tumor cells at irregular mucosa. **(C)** Pathologic examination of the biopsy sample obtained from the irregular mucosa revealed carcinoma *in situ* of bladder. pCLE, probe-based confocal laser endomicroscopy.

After removing the Foley catheter and replacing the rigid cystoscope with a 12-degree lens, pCLE imaging was performed using a pCLE system with a 2.7 mm cystoflex-UHD Cellvizio fiber (Cellvizio; Mauna Kea Technologies, Paris, France), which was inserted using the working channel of the cystoscope. The laser probe was placed directly adjacent to the region of interest. pCLE detected the nuclei of cancer cells within the irregular mucosa in the bladder diverticulum. After observation using pCLE, mucosal biopsy and transurethral resection (TUR) were performed. Biopsy revealed bladder CIS (Fig. 1). After TUR, this patient underwent Bacillus Calmette-Guerin therapy.

## Discussion and Literature Review

Photodynamic diagnosis (PDD) or narrow band imaging (NBI) is useful for the detection of CIS.^5,6^ We compared PDD with NBI in the same patients with flat urothelial lesions and describe the efficacy of the combined use of PDD and NBI for the detection of bladder CIS.^7^ In doing so, we found that PDD or NBI both have a lower specificity for bladder UC detection than white light cystoscopy.

Recently, several authors have reported the efficacy of pCLE for the detection of bladder UC using fluorescein.^1,2^ Given the limited visual field and depth, pCLE cannot be used to completely explore the bladder. Nevertheless, pCLE can be used to obtain histologic images of the targeted areas. pCLE can also provide histologic information about the tumor area and edges. The natural fluorescence of tissues without a fluorescent dye is insufficient for pCLE. Fluorescein is approved by the Food and Drug Administration, and has been used in clinical practice for many years by gastroenterologists; however, it cannot be used to stain the cytoplasm and nuclei of cells. Liu et al. reported the efficacy of acriflavine for CLE in patients who underwent upper gastrointestinal CLE.^3^ However, acriflavine is harmful to the eyes and mucosa if inhaled, and causes skin irritation. Meanwhile, we have reported that pCLE using acrinol can be used to detect the nuclei of UC in an *ex vivo* study.^4^ Acrinol, which is a derivative of acriflavine, can be used as an antiseptic drug in the urologic, gynecologic, and dermatologic fields in Japan. In this case, we were able to observe the cell nuclei of bladder CIS that could not be detected using conventional cystoscopy with white light. pCLE using acrinol can be used as an optimal biopsy of bladder CIS during TUR. Since this study is a case report, a further prospective study will be needed to determine the efficacy of NBI or PDD in detecting bladder cancer.

## Conclusion

pCLE using acrinol as a novel dye can be used to observe the nuclei of bladder carcinoma tissue *in situ*, which otherwise cannot be detected using conventional cystoscopy under white light. pCLE using acrinol is a potential modality for performing an optical biopsy during TUR.

## Abbreviations Used

CIS: carcinoma *in situ*
CT: computed tomography
MRI: magnetic resonance imaging
NBI: narrow band imaging
pCLE: probe-based confocal laser endomicroscopy
PDD: photodynamic diagnosis
TUR: transurethral resection
TURBT: transurethral resection of a bladder tumor
UC: urothelial carcinoma
